# Cholera as It Appeared in Nashville

**Published:** 1849-05

**Authors:** A. H. Buchanan


### WESTERN JOURNAL

OF

### MEDICINE AND SURGERY.

MAY, 1849.

### Art. I.—*Cholera as it appeared in Nashville.* By A. H. Buchanan, M.D., in a letter to Prof. Yandell.

Dear Sir:—On the 27th of December, 1848, the steamer Caroline Watkins, from N. O., where cholera was prevailing when she left that port, arrived in Nashville one death having occurred on board from cholera. On her trip up to this city, occupying a period of about ten days, she lost eight persons from the same disease. These deaths occurred among deck passengers and firemen, most of whom were foreigners, and, as represented, very imprudent under treatment; one or two of the deaths were among colored persons. On her arrival in our city great excitement prevailed among the citizens, and every one, in anticipation of the appearance of the disease amongst us, was anxious to learn, each from his own medical adviser, what must be done to prevent or cure its dreaded attack. Various prescriptions were made, each physician prescribing according to his own notions of its pathology and treatment. The apothecaries were busy in supplying families with the means under the advice of their physicians, and sometimes without, to combat this fearful malady. The city authorities were also awake to its approach, and every effort was made (I am happy to say) effectually to cleanse the city in anticipation of its appearance. Thus, were we prepared, and fully prepared, for an attack. Almost every family had the remedies at hand, and were well instructed how to use them at a moment's warning. But for three weeks there was no appearance of cholera, and the excitement began to subside, when on the 20th of January, twenty-five days after the arrival of the Watkins, and several days after the arrival of other boats on board of which there was cholera, one case occurred in the lower part of the city, in a house almost surrounded by water; for at this time there was a great flood in the Cumberland, so that the upper and lower parts of the city were inundated.*

The occupants of the house in which this case occurred were of the lowest and most indigent class. This case, a man of intemperate habits, and who had exposed himself to the weather and in the water for several days, died in a few hours after his attack.

On the next day, the 21st, another inmate of this house, and a fellow in all respects to the first case, was seized with the disease but was immediately removed to comfortable quarters, where, under good medical advice and nursing, he seemed to be convalescent, but in a few days died from his own imprudence. The third case

---

* Nashville descends from the center on the banks of the Cumberland both up and down the river.

occurred in the same house; another associate of the two first was seized on the 22d, and was also removed, together with all the families who occupied the house, to better quarters in a more elevated part of the city. But notwithstanding every effort was made to save him, he collapsed, and died in about thirty-six hours. This case I visited in its last stages, and recognized all the characteristic symptoms of Asiatic cholera, such as I had before witnessed in 1833–'34. The haggard but indifferent countenance, cold, blue, and shriveled extremities, pulseless wrist, sunken, or almost lost voice, with clammy sweat, were all present; as also rice-water evacuations, which clearly indicated the nature of the disease. From this time cholera gradually increased; several more deaths occurred in this family and in the immediate neighborhood. It also made its appearance in the upper part of the city, among a class of citizens much more comfortably situated and greatly above high water mark. The force of the disease seems to have been spent upon these two neighborhoods, but several deaths occurred in the heart of the city, and a few of our most respectable citizens were carried off by it. One family, in which the disease appeared with great severity, lived in a good house in an open lot or field, upon an elevated situation in the suburbs of the city and seemed to be remote from all city influences, or other causes productive of it. In this family two deaths occurred and every member suffered from it. The black population and the lower classes of society, the most ignorant and indigent, were the greatest sufferers.

About the 4th or 5th of February the disease reached its greatest height and as many as four or five died in twenty-four hours. Previous to this time from one to two orthree deaths occurred daily, and after this the disease gradually declined, so that by the 26th Feb. we regarded it as at an end. The number of deaths were in all about forty or fifty.

During the prevalence of the disease almost every one in the city complained more or less of some uneasiness in the stomach and bowels. Hundreds suffered from diarrhea and some from dysentery, which in a few cases was very painful and difficult to cure. Many were afflicted with violent spasms of the stomach and bowels and abdominal muscles without discharges, similar to what we commonly call cramp colic. In some cases all the voluntary muscles were violently cramped; the spasms recurred at intervals of a few minutes, not unlike tetanus. Several cases of typhoid fever occurred during its prevalence but the symptoms were not influenced by it in the slightest degree. In the month of December and until late in January our community all suffered more or less from a mild epidemic influenza. Only in a few instances did it appear in a violent form or require medical aid. When cholera made its appearance it rapidly subsided, and very rarely did we witness a complication of choleric symptoms with those of influenza.

You are well aware that several of our physicians here regard cholera as contagious, and many of the most venerable of the profession will not admit of the existence of a case, without it can be traced to a contagious source, nor do they regard the diarrhea or other disordered conditions of the stomach and bowels as they have prevailed here as at all premonitory of cholera. I differ very, very widely from those who entertain such opinions and think we have passed through an epidemic form of cholera, which, compared to the epidemic of 1833 and 1834, was mild in its character, but which, except for the prompt and efficient means used to control it, would nevertheless have proved much more fatal than it really did. I considered the whole community as more or less affected by it and advised all who consulted me to regard every symptom of disorder in the digestive organs, especially a diarrhea as premonitory of an attack, or in other words as the commencing or first stage of cholera, and to use means at once to arrest it. Such also, I believe, was the opinion and advice of a majority of the physicians in our city. Here then are the causes of the boasted success in the treatment of the late epidemic.

1st. Our city was well cleansed and in good condition when it appeared.

2d. Every family who were able were supplied with remedies, and were aware of the nature of the first symptoms of the disease, and also of the best modes of living to prevent an attack.

3d. The epidemic was mild in its character and gave all fair warning, so much so, that even those who died might almost certainly have been saved had their intelligence and means been sufficient to guard against its insidious attack. It is well ascertained that all those who died had diarrhea or other premonitory symptoms for two or three days or longer before they applied for assistance. And some cases were even in a collapsed state before medical advice was sought. But very few, if any cases occurred suddenly and violently, as in the former epidemic. All those who applied to a physician in the first stages of diarrhea were promptly relieved, and hundreds cured themselves with the remedies at hand, with which they had been previously furnished and instructed how to use. Cholera then as it lately appeared in Nashville was certainly a very curable disease. Taken in time it was curable in almost every instance.

You will perceive from the annexed meteorological diary, which was furnished by the Hon. Judge M. W. Brown, for January and February, that the weather was neither very cold nor warm for the season. The thermometer ranged in January from 13°, on the 6th, to 68° on the 30th, and was generally about freezing point at sunrise, and 35° or 40° in the afternoon. But, for the most part the weather was exceedingly disagreeble, being moist, cloudy and chilly, with wet and muddy streets and with a flood in the river which reached its maximum height on the 22d, being 45½ feet above low water mark. The 3d violent case of cholera occurred on the 22d, the day on which the flood was at its height.

The winds during this month were variable, but prevailed from the N. N. E. and S. S. E. The day on which the first case occurred was cloudy and drizzling rain. Thermometer 40° at sunrise, wind S. and S. W. The balance of this month was cloudy and rainy with a temperature generally above freezing point at sunrise, and from 40° to 60° in the afternoon. The weather during the month of February was similar to January, but was colder and clearer and the winds prevailed from the N. N. W. We had several beautiful days in February, with bracing winds and clear sky. The middle of the month was very cold. Thermometer on the 18th being 4° and on the 10th ice formed on the ponds three and four inches thick, and our ice-houses were all filled. From this time the weather grew warmer, and on the 26th cholera left our city. At present we are entirely free from the disease. The adjoining counties, as I learn from letters received, have suffered in a slight degree from diarrhea and general disorder of the digestive organs. You have now a pretty correct idea of the rise, progress and termination of cholera in our city. But before closing my remarks I must say a few words on the treatment.

In all cases of premonitory symptoms, such as pain and fulness in the stomach, which was often complained of, and in diarrhea which was the most common symptom, I advised the use of the following mixture:

Take—Alcohol - - - - - - - - 1 pint,
Gum Camphor - - - - - ℥ij

Dissolve and add

Laudanum - - - - - - ℥ij
Compound spirits Lavender ℥ij

Of this mixture 30 to 60 drops were directed to be used upon a lump of loaf sugar, and repeated after each evacuation from the bowels. Or in case of pain or spasm in the stomach or bowels, every half hour until relief was obtained. In many violent cases it was used in larger doses, and in cases of mere uneasiness in doses of 10 or 20 drops repeated pro re nata, as a preventive. I assure you it had the desired effect in all cases in which it was used, and hundreds cured themselves with it who suffered from diarrhea without the aid of any other remedy. In more violent cases or where the diarrhea and vomiting were suffered to run on without treatment until spasms in the muscles, prostration and approaching collapse were evident, I used in addition to the above 2 or 3 gr. doses of opium and repeated at intervals of one, two, or three hours, according to the urgency of the symptoms. The pill of opium was generally washed down with brandy and water, and as many as 10 or 12 grains were administered in bad cases in twenty-four hours. In the absence of opium, laudanum was used with the brandy in teaspoonful doses, and in very urgent cases of spasms and approaching collapse, all these remedies, together with sinapisms to the abdomen and extremities, and also hot applications to the feet and stomach, and other parts suffering from cramps and pain were applied. The patient all the while being well covered up in bed and using hot sage, mint, or other teas, such as were most agreeable. This course of treatment was successful in every case that came under my own observation, except in two cases, and one of these was collapsed at my first visit. In no case did I use one grain of calomel or other mercurial, except in two instances, and then I believe with disadvantage. In the large majority of cases the bowels were checked up at once by the above treatment and were suffered to remain so for three or four days without evacuations. At the end of this time, or earlier in case of pain or other disposition to stool without evacuations, a tablespoonful of castor oil with 15 or 20 drops of laudanum generally produced a free bilious discharge, such as often follows a dose of calomel, and left the patient convalescent.

In some cases however, this prostration is followed by very slow recovery, or terminates in death. A low typhoid state of fever comes on with red and swollen tongue, feeble voice, sore throat, dry fauces, sighing, nausea, &c. which is always critical. Such cases require the most judicious treatment, and even under the best directed skill will often terminate fatally. But I cannot enter into details as regards the treatment of such cases. Your patience must be already worn out in the perusal of this long letter. I will only observe before closing that I have no prejudice against the use of calomel in the treatment of cholera or any other disease. On the contrary it is my favorite remedy in the treatment of almost all diseases. I merely wish to state a fact as observed in connection with the late epidemic, which naturally gives rise to the reflection that, possibly, we place too much confidence in its powers in other diseases. I confess I was surprised to see free bilious discharges without the use of some mercurial. But such was the fact in many instances from a dose of oil, and in many cases without the aid of any purgative, and where the bowels had been bound up for 2 or 3 days by the use of opiates, the first evacuations that followed were bilious and the patients rapidly convalesced. Should the epidemic unfortunately reappear amongst us during the spring and summer a more extended series of observations on these points will be interesting and possibly I may be heard from on the subject again.

Appended is the meteorological table to which I have referred in this communication.

| DATE | REMARKS. | THERM. mi | max | BAR. | WIND. |
|---|---|---|---|---|---|
| M. 1. | Frost, Fog. Partially cloudy. D. P. 30 | 29 | 55 | 29.60 | Mod. w.s.w. |
| T. 2. | Partially cloudy..........D. P. 28 | 34 | 42 | 29.70 | Brisk n. e. |
| W. 3. | Cloudy, snowed, with intermission, drizzling of rain in eve and night D. P. 25 | 30 | 32 | 29.50 | Mod. n. e. and e.n.e. |
| T. 4. | Cloudy, cold.............D. P. 20 | 26 | 30 | 29.56 | Mod. n. |
| F. 5. | Cloudy, clear at night.....D. P. 13 | 23 | 26 | 29.80 | Mod. n. more brisk [eve |
| S. 6. | Generally clear, frost, slightly cloudy at night..............D. P. 14 | 13 | 32 | 29.88 | Generally calm. |
| S. 7. | Snowed from 4 to 10½ a. m., cloudy rest of the day..........D. P. 25 | 26 | 36 | 29.60 | Mod. e.—s.e. and s. |
| M. 8. | Cloudy.................D. P. 20 | 32 | 38 | 29.55 | Mod. brisk n.e. |
| T. 9. | Cloudy and snowed for an hour in forenoon, partially clear in eve ....................D. P. 20 | 30 | 29 | 29.55 | Brisk, n. |
| W 10 | Partially cloudy clear at night...... ....................D. P. 18 | 22 | 28 | 29.87 | Mod. n.n.w. |
| T. 11 | Clear, frost; partially cloudy in afternoon.................D. P. 18 | 16 | 36 | 29.90 | Mod. e.n.e. & e.s.e. |
| F. 12 | Partially cloudy in forenoon—overcast in the afternoon, rain at 8 at night continued........D. P. 34 | 29 | 42 | 29.80 | Gusty from s.e. & s |
| S. 13 | Cloudy; rain set in at night and continued very brisk......D. P. 37 | 42 | 51 | 29.55 | Gusty s.w. & s. |
| S. 14 | Continued rain until night, after night ther. rose suddenly from 40 to 58, wind gusty from s. at the time and some lightning D. P. 37 | 37 | 37 | 29.66<br>—40 | n. n.e. & e. s. |
| M. 15 | Storm before day, heavy wind s w with some thunder and lightning and continued rain to 8 a m. Wind having changed to n w, partially clear at night..D. P. 37 | 48 | 40.58<br>50 | 29.64 | Brisk n.w. |
| T. 16 | Frost, partially cloudy....D. P. 32 | 32 | 50 | 29.75 | e. & s.e after night [gusty from s. |
| W 17 | Heavy rain before day with thunder rained to 7 a m........D. P. 38 | 48 | 50.46 | 29.60 | n.w. & n. |
| T. 18 | Lightly overcast in forenoon, generally clear in afternoon and night ....................D. P. 22 | 30 | 29 | 30.10 | Mod. n. e. |
| F. 19 | Frost, clear generally.....D. P. 28 | 22 | 40 | 30.05 | Mod. e. & e.s.e. |
| S. 20 | Cloudy and drizzle of rain D. P. 33 | 40 | 44 | 29.82 | Mod. s. & s.s.w. |
| S. 21 | Overcast and some rain, clouds thin in evening and broken..D. P. 32 | 48 | 45 | 29.76 | Brisk n.w. & n. |
| M. 22 | Thinly overcast in forenoon generally clear in the evening and at night.................D. P. 27 | 30 | 33 | 29.95 | Mod. n.w. & n. |
| T. 23 | Slightly overcast in morn, clear after generally...........D. P. 28 | 27 | 41 | 29.80 | Mod. e. |
| W 24 | Cloudy.................D. P. 50 | 35 | 58 | 29.66 | Mod. s. e. & s. gusty at night |
| T. 25 | Cloudy and brisk rain at 2½ p m to 5. Clouds broken after, lingtning at night................D. P. 55 | 59 | 64 | 29.48<br>38.— | Gusty s.s.w. & s. |
| F. 26 | Cloudy, clouds light in evening ....................D. P. 33 | 37 | 44 | 29.66 | Gusty before day [mod. after n.w. |
| S. 27 | Slightly overcast in forenoon, clear after.................D. P. 31 | 33 | 44 | 29.74 | Mod. n.e. & e. |
| S. 28 | Generally clear..........D. P. 40 | 35 | 60 | 29.67 | Mod. s. [s.w. |
| M. 29 | Generally cloudy.........D. P. 46 | 54 | 60 | 29.60 | Mod. brisk, s.s.w. & |
| T. 30 | Cloudy, slight rain at 3 p m and at night brisk shower......D. P. 52 | 62 | 68 | 29.50 | Brisk s.w. changed [to n.w. at 3 pm |
| W 31 | Cl'dy, rain before day, partilly clear at night for an hour or two. D. P. 44 | 44 | 58 | 29.54<br>40— | Mod. n. & n.e. ch [to s. at nt. & gus |

| DATE | REMARKS. | THERM. mi | THERM. max | BAR. | WIND. |
|---|---|---|---|---|---|
| T. 1 | Rain before day and storm from n.w. partially clear, brisk rains again at night..................D. P. 48 | 56 | 60 | 29.51 43 | Mod. nw & n gen. |
| F. 2 | Rain before day and until 7 a m, cloudy rest of the day....D.P. 30 | 52 | 42 | 29.61 | Mod. brisk nw & n |
| S. 3 | Generally clear..........D. P. 33½ | 29 | 44 | 29.80 | Mod. n & ne |
| S. 4 | A little sleet in the morn, after rain more brisk in evening, snow at night | 37 | 40 | 29.56 34 | Brisk ne & n |
| M. 5 | Clear—light cumuli from n.w. ......................D. P. 24 | 30 | 34 | 29.60 | Brisk nw. [-nw |
| T. 6 | Generally clear...........D. P. 23 | 28 | 46 | 29.55 | Gusty wsw & wwnw |
| W. 7 | Generally clear...........D. P. 20 | 20 | 36 | 29.83 | Mod nw & ssw |
| T. 8 | Mod. rain at times.........D. P. 30 | 38 | 42 | 29.59 | Brisk sw at night nw |
| F. 9 | Clear.....................D. P. 22 | 28 | 44 | 29.64 | Mod. nw |
| S. 10 | Frost, clear..............D. P. 18 | 24 | 50 | 29.60 | Mod. n. |
| S. 11 | Clear, except eveing slightly overcast.....................D. P. 25 | 26 | 56 | 29.34 | [eve br nw Mod. & var in m, in |
| M. 12 | Clear.....................D. P. 20 | 28 | 53 | 29.48 | Brisk n |
| T. 13 | Generally clear...........D. P. 23 | 25 | 53 | 29.62 | Mod. brisk nw |
| W 14 | Cloudy, snowed at 2½ until night clear after night.........D. P. 23 | 34 | 26 | 29.78 | Brisk nw |
| T. 15 | Generally clear...........D. P. 1½ | 13 | 28 | 29.78 | Mod. nw |
| F. 16 | Frost, clear—cloudy to the west in the evening.............D. P. 12 | 10 | 33 | 62 29.48 | Mod. sw |
| S. 17 | Overcast, thinly in forenoon and a little snow; heavy snow squalls in afternoon with intervals of partial clearness, continued at night and becoming very cold..D. P. 20 | 25 | 34–24 | 29.26 | Gusty from w & nw |
| S. 18 | Generally clear.............D. P. 4 | 4 | 24 | 29.88 | Brisk nw |
| M. 19 | Frost, clear except evening slightly overcast.................D. P. 10 | 7 | 32 | 30 | Mod. e |
| T. 20 | Cloudy, a little sleet morning, drizzle of rain after.........D. P. 24 | 33 | 34 | 29.72 | Mod. e & se |

